# Antibiotic Resistance to *Pseudomonas Aeruginosa* in the Saliva of Candidates of Bone Marrow Transplantation

**DOI:** 10.30476/dentjods.2024.100492.2225

**Published:** 2025-03-01

**Authors:** Maryam Zahed, Mojtaba Safvat, Abdollah Bazargani, Janan Ghapanchi, Zahra Ranjbar

**Affiliations:** 1 Oral and Dental Disease Research Center, Dept. of Oral and Maxillofacial Medicine, School of Dentistry, Shiraz University of Medical Sciences, Shiraz, Iran; 2 Undergraduate Student, Student Research Committee, School of Dentistry, Shiraz University of Medical Sciences, Shiraz, Iran; 3 Dept. of Bacteriology and Virology, School of Medicine, Shiraz University of Medical Sciences, Shiraz, Iran; 4 Dept. of Oral and Maxillofacial Medicine, School of Dentistry, Shiraz University of Medical Sciences, Shiraz, Iran

**Keywords:** Drug Resistance, Hematopoietic Stem Cell Transplantation, *Pseudomonas aeruginosa*, Saliva

## Abstract

**Statement of the Problem:**

Opportunistic infections such as *Pseudomonas aeruginosa*, colonized from the oral cavity, are common in candidates of bone marrow transplantation. Therefore, it is important to prescribe appropriate antibiotics for this group of debilitated patients.

**Purpose:**

In this study, we aimed to investigate the antibiotic resistance to *Pseudomonas aeruginosa* encountered in candidates of bone marrow transplantation referred to Imam Reza Clinic, Namazi Hospital, and Amir Hospital, Shiraz, Iran.

**Materials and Method:**

This cross-sectional study was performed on 30 patients with leukemia and lymphoma. To identify Pseudomonas species, saliva samples were collected and cultured in a blood agar medium. Considering the present therapies, we examined the antibiotic sensitivity of Penicillin, Oxacillin, Gentamicin, Tetracycline, Cefepime, Ciprofloxacin, Imipenem, Ceftazidime, Cefotaxime, Erythromycin, Trimethoprim, Sulfamethoxazole, and Vancomycin. Statistical analysis was performed using chi-square test (χ^2^) and SPSS software. The *p* Value< 0.05 was considered significant.

**Results:**

In 10 patients out of 30 for whom *Pseudomonas aeruginosa* was found positive (33.33%), all cultures were resistant to Penicillin and Oxacillin (100%), followed by Trimethoprim and Sulfamethoxazole (80%). The best performance was seen by Vancomycin (20%). No correlation was found in terms of age, gender, and type of disease (lymphoma or leukemia) for antibiotic resistance (*p*> 0.05).

**Conclusion:**

It seems that Penicillin, Oxacillin, Tetracycline, Sulfamethoxazole have significant resistance to Pseudomonas aeruginosa. Further research on Gentamicin, Ceftazidime, Ciprofloxacin, and especially Vancomycin is recommended for effective antibiotic prescriptions against *Pseudomonas aeruginosa*.

## Introduction

Cancers are one of the biggest global health problems after cardiovascular disease, traumatic events, and major crises in the world [ 1 ]. The prevalence of leukemia and lymphoma in Iran is significantly high [ 2 ]. Acute leukemia is responsible for more than 10,000 deaths per year despite improved treatment regimens and new cytostatic drugs [ 3 ] and infection is one of the most serious complications and the leading cause of death in patients with acute leukemia [ 4 - 5 ]. Opportunistic infections are common in individuals with leukemia and lymphoma due to immunodeficiency caused by immature leukocytes or chemotherapy and the use of broad-spectrum antibiotics [ 6 - 7 ]. In the early stages of leukemia and lymphoma, leukopenia may occur as a result of bone marrow suppression and extensive proliferation of white blood cells (WBCs). Agranulocytosis, pancytopenia, and leukopenia also occur, which are commonly the result of drug toxicity. In addition, cyclic neutropenia, a periodic decrease in neutrophils, is also seen in association with leukemia, lymphoma, or drug therapy [ 6 ].

*Pseudomonas aeruginosa* (*P. aeruginosa*) is present in the normal flora of the intestine and the surface of human skin and it is an important pathogen in these locations. It is an opportunistic bacterium involved in a variety of acute and chronic urinary tract infections, respiratory system infections, inflammation of the skin, soft tissue infections, bacteremia, bone and joint infections, various gastrointestinal infections, and systemic infections, especially in patients with severe burns. This bacterium is mainly found in people with host defense deficiencies such as cancer, human immunodeficiency virus (HIV) and cystic fibrosis (CF) and it is a significant cause of death in such patients [ 8 ]. This microorganism is also abundant in a variety of water resources, especially in environments such as hospitals [ 9 ] and health care centers [ 9 - 10 ]. It is shown that the oral cavity can be a reservoir for *P. aeruginosa* in debilitated patients [ 10 - 11 ]. Tonsillar crypts, tongue, and gingiva can be considered a reservoir for this bacteria [ 11 - 12 ]. Moreover, patients positive with subgingival *P. aeruginosa* are reported to show a higher percentage of periodontal sites with suppuration on probing, greater periodontal attachment loss, and much more aggressive forms of periodontitis [ 11 ]. 

Infections caused by this pathogen are often difficult to treat due to multiple drug resistance and sometimes, more advanced antibiotic drug regimens are required leading to numerous side effects. In fact, over the past few years, *P.aeruginosa* has shown increasing resistance to many antimicrobial drugs, including Carbapenems and β-lactam antibiotics that are widely used in clinical practice [ 13 ]. Moreover, not only prophylactic treatments such as mouthwashes do not sufficiently reduce the bacterial load in the mouth and larynx but also they cannot prevent aspiration pneumonia in at-risk patients, especially in cases with underlying pathologies such as blood malignancies [ 14 ]. Therefore, oral examination is still necessary to improve treatment strategies, especially for patients with hematologic malignancies.

Previous studies have shown an increase in *P. aeruginosa* resistance to routine antibiotics [ 10 , 15 - 17 ], especially Penicillin [ 15 ]. Ten-year cross sectional studies have also shown an increase in resistance to antibiotics such as Co-amoxiclav (Amoxicillin / Clavulanic acid), Tetracycline, and Imipenem [ 18 - 19 ].

Since patients with leukemia and lymphoma are prone to opportunistic infections such as *P.aeruginosa*, and given that this bacterium can be isolated from the saliva, this study aimed to evaluate the antibiotic resistance of *P. aeruginosa* in the saliva of candidates of hematopoietic stem cell transplantation (HSCT) in Shiraz, Iran. It is important to prescribe appropriate antibiotics to prevent antibiotic resistance in these patients.

## Materials and Method

This cross-sectional study was performed in May-December 2019 on 30 patients with a history of leukemia or lymphoma, candidates of bone marrow transplantation referred to Imam Reza Clinic, Namazi Hospital, and Amir Hospital, Shiraz, Iran. In the study group, 30 patients with history of lymphoma and leukemia were included after signing an informed consent form. The sample size was chosen according to a previous study [ 20 ]. All patients had finished their chemotherapy course and they were not on any medications at the time of the study. Patients who had not used systemic corticosteroids for the past 2 months or topical corticosteroids for the past 2 weeks were included. Patients with diabetes, acquired immunodeficiency syndrome (AIDS), systemic diseases affecting the immune system (including autoimmune diseases and thyroid diseases), and patients who had used mouthwashes and antibiotics in the past month were excluded from the study. 

### Ethical Considerations

The study was in accordance with the *declaration of Helsinki* and approved by the Ethics Committee of Shiraz University of Medical Sciences. (IR.SUMS. DENTAL.REC.1399.124)

### Sample Collection

Participants were abstained from eating and drinking for half an hour before sampling. A total of 3cc of non-stimulating saliva was collected. Their saliva samples were sent to the microbiology laboratory in a transport environment through a cold chain. Samples were cultured in blood agar medium and incubated at 37°C for 24 hours. To separate *P. aeruginosa* blood agar culture, gram staining, catalase and oxidase tests were used.

### Microscopic Gram Staining and Biochemical Tests

The microscopic examination of the bacterial isolates cells was carried out using an inoculation loop to transfer a part of a young colony. The colony was mixed with a drop of water and then spread on the surface of a slide and left to dry, then fixed with heat over a gentle flame and stained with gram stain and examined under the microscope where the shapes and arrangement of bacterial cells were observed. In addition, the colors that result from the interaction of bacteria with the dye were examined.

Biochemical tests were done, such as the catalase test to determine the ability of the isolates to produce the catalase enzyme that breaks down hydrogen peroxide into water and oxygen gas, and the oxidase test to evaluate the ability of bacterial colonies to produce the oxidase enzyme. These tests were all conducted according to standard methods [ 21 ]. 

### Antibiotic Resistance

To determine the pattern of antibiotic susceptibility and resistance of Pseudomonas isolates, a disk diffusion test (Kirby-Bauer) was used. Considering the present therapies, a variety of antibiotics including Penicillin, Oxacillin, Gentamicin, Tetracycline, Cefepime, Ciprofloxacin, Imipenem, Ceftazidime, Cefotaxime, Erythromycin, Trimethoprim, Sulfamethoxazole, and Vancomycin were examined to determine antibiotic sensitivity. The microbial suspension was first prepared for all collected strains and then cultured on Mueller-Hinton agar medium (Merck, Germany) with a sterile swab, after which antibiotic discs were implanted with sterile forceps. The plates were incubated at 35°C for 24-48 hours. Then the diameter of growth halos was measured with a ruler and its interpretation was performed according to the recommendations of the Clinical and Laboratory Standard Institute (CLSI) table. Disks quality control was checked using ATCC 27853 strain [ 2 ].

### Statistical Analysis

Statistical analysis was performed by chi-square test (χ^2^) with Statistical Package for the Social Sciences (SPSS Inc.; Chicago, IL, USA) version 18. The results were presented in the form of descriptive statistics in terms of relative frequency. The correlation analysis was based on *p* Values, and *p* Value < 0.05 was considered significant.

## Results

**Figure 1:**
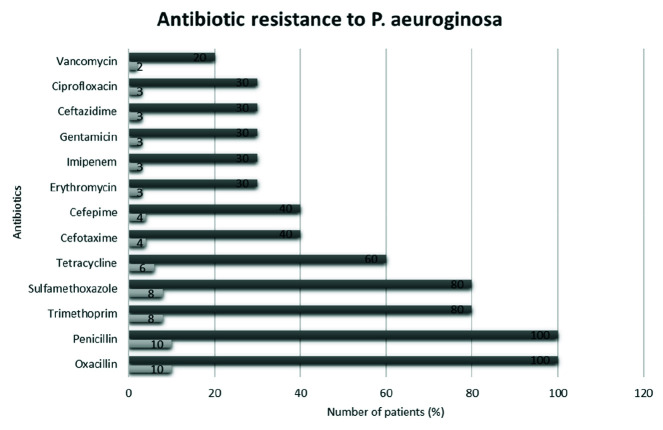
The distribution pattern of *P. aeruginosa* antibiotic resistance in candidates of bone marrow transplant

From 30 bone marrow transplant candidates, 12 were suffering from lymphoma and 18 were leukemic patients. 10 cases (4 lymphomas and 6 leukemic patients; 33.33%) were positive for *P. aeruginosa* and their resistance pattern was evaluated. The pattern of *P. aeruginosa* antibiotic resistance in all patients is presented in Figure 1. *P. aeruginosa* was resistant to Penicillin and Oxacillin (100%) in all cases, followed by Trimethoprim and Sulfamethoxazole (80%). The best performance was seen in Vancomycin (20%).

Antibiotic resistance in relation to age, gender, and type of disease is presented in Tables 1-3, respectively. According to Table 1, there was no significant relationship between age and antibiotic resistance of this bacteria (*p*> 0.05). In the case of Penicillin and Oxacillin, all patients were resistant to these antibiotics. Concerning gender, there was no significant relationship between this variable and antibiotic resistance of *P. aeruginosa* (*p*> 0.05) (Table 2). In addition, there was no significant relationship between the type of disease and antibiotic resistance of the bacteria (*p*> 0.05) (Table 3).

**Table 1:** The distribution pattern of *P. aeruginosa* antibiotic resistance by age in bone marrow transplant patients

Antibiotics	Age of each patient														*p* Value (chi square)
	8		11		14		23		30		46		64		
	N	(%)	N	(%)	N	(%)	N	(%)	N	(%)	N	(%)	N	(%)
Erythromycin	0	0%	0	0%	1	10%	1	10%	1	10%	0	0%	0	0%	0.267
Oxacillin	2	20%	1	10%	1	10%	1	10%	2	20%	1	10%	2	20%	-
Imipenem	1	10%	1	10%	0	0%	0	0%	0	0%	0	0%	1	10%	0.514
Penicillin	2	20%	1	10%	1	10%	1	10%	2	20%	1	10%	2	20%	-
Tetracycline	0	0%	0	0%	1	10%	0	0%	2	20%	1	10%	2	20%	0.125
Trimethoprim	2	20%	1	10%	1	10%	1	10%	1	10%	0	0%	2	20%	0.333
Gentamicin	1	10%	0	0%	1	10%	0	0%	0	0%	0	0%	1	10%	0.514
Ceftazidime	1	10%	0	0%	0	0%	0	0%	1	10%	0	0%	1	10%	0.827
Cefotaxime	0	0%	1	10%	0	0%	0	0%	2	20%	0	0%	1	10%	0.244
Cefepime	0	0%	1	10%	0	0%	0	0%	2	20%	0	0%	1	10%	0.442
Sulfamethoxazole	2	20%	1	10%	1	10%	1	10%	1	10%	0	0%	2	20%	0.333
Ciprofloxacin	1	10%	0	0%	0	0%	1	10%	0	0%	0	0%	1	10%	0.514
Vancomycin	0	0%	1	10%	0	0%	0	0%	1	10%	0	0%	0	0%	0.333
Total Number of patients in each group of age	2	20%	1	10%	1	10%	1	10%	2	20%	1	10%	2	20%	Total 10 (100%)

**Table 2:** The distribution pattern of *P. aeruginosa* antibiotic resistance by gender in bone marrow transplant patients

Antibiotics	Gender				*p* Value (chi square)
	Male		Female		
	N	(%)	N	(%)	
Erythromycin	1	10%	2	20%	0.490
Oxacillin	5	50%	5	50%	-
Imipenem	2	20%	1	10%	0.490
Penicillin	5	50%	5	50%	-
Tetracycline	2	20%	4	40%	0.197
Trimethoprim	4	40%	4	40%	1.000
Gentamicin	1	10%	2	20%	0.490
Ceftazidime	2	20%	1	10%	0.490
Cefotaxime	3	30%	1	10%	0.197
Cefepime	2	20%	2	20%	1.000
Sulfamethoxazole	4	40%	4	40%	1.000
Ciprofloxacin	1	10%	2	20%	0.490
Vancomycin	2	20%	0	0%	0.114

**Table 3:** The distribution pattern of *P. aeruginosa* antibiotic resistance by disease type in bone marrow transplant patients

Antibiotics	Disease			
	Lymphoma		Leukemia	
	N	(%)	N	(%)
Erythromycin	1	10%	2	20%
Oxacillin	4	40%	6	60%
Imipenem	2	20%	1	10%
Penicillin	4	40%	6	60%
Tetracycline	1	10%	5	50%
Trimethoprim	4	40%	4	40%
Gentamicin	2	20%	1	10%
Ceftazidime	1	10%	2	20%
Cefotaxime	1	10%	3	30%
Cefepime	1	10%	3	30%
Sulfamethoxazole	4	40%	4	40%
Ciprofloxacin	1	10%	2	20%
Vancomycin	1	10%	1	10%

## Discussion

This study aimed to evaluate the pattern of *P. aeruginosa* antibiotic resistance among bone marrow transplant patients. The results showed no significant relationship between the three factors of age, gender, and type of disease (leukemia and lymphoma) for the pattern of *P. aeruginosa* antibiotic resistance among bone marrow transplant patients. However, a complete resistance of Penicillin and Oxacillin was observed against this bacterium. Cefepime and Trimethoprim had 80% resistance and Vancomycin showed the best performance with only two cases (20%) of resistance.

Resistance to antimicrobials is a serious problem for human health worldwide. This has been associated with increased patient mortality, delays in hospitalization, and increased costs [ 22 ]. In recent years, this problem has arisen due to the rapid proliferation of Metallo-β-lactamases that mediate resistance to Carbapenems (such as Imipenem) among gram-negative bacilli [ 23 ]. In 2017, for the first time, the World Health Organization (WHO) published a list of drug-resistant bacteria that were in atrocious need of new antibiotics. In this list, *P. aeruginosa* was ranked at the critical level having resistance to Carbapenem. Antibiotic resistance kills almost 700,000 people a year worldwide and some experts predict that this number will reach to 10 million by 2050 [ 24 ].

A 2019 study by Jarlier *et al.* [ 15 ] showed an 8.9% prevalence of *P. aeruginosa* in 30 different countries, with significant percentages of resistance to third-generation Cephalosporins, Carbapenems, and Fluoroquinolones. In another study, it is shown that patients who were infected more than once with *P. aeruginosa* were younger than those who were infected only once. These patients spent more days in the hospital and the intensive care unit (ICU). They underwent biopsy and transplantation; they were exposed to invasive medical devices, and were diagnosed with cystic fibrosis and kidney disease. They also took antibiotics other than Penicillin for longer periods [ 16 ]. In addition, more recurrences of gram-negative bacterial infections in the hospital were associated with a higher risk of drug-resistant infections [ 17 ]. For patients with recurrent *P. aeruginosa* infections, the risk of drug resistance was increased by 23% compared to any previous hospital infection. Moreover, approximately 15% of these infections became drug-resistant afterwards and 44% remained drug-resistant [ 17 ]. 

Besides, infection with gram-negative bacteria is found to be common after hematopoietic stem cell transplantation (HSCT) and organ transplantation and it is associated with high mortality rates [ 24 - 25 ]. Infections can also affect the life quality of such patients [ 26 - 27 ]. In Satlin and Walsh’s study 2017 [ 28 ], the results showed that *P. aeruginosa* was increasingly found in the bloodstream of HSCT patients and the prognosis of subjects infected with these bacteria was worse than patients infected by more susceptible bacteria. 

Concerning the history of antibacterial resistance for this bacterium, we can refer to the study of Ramos *et al.* [ 18 ], which was conducted in Brazil over a period of 10 years (1998-2008). This study examined the presence of Pseudomonas resistance to Tetracycline and β-lactams in the oral cavity of patients with gingivitis, periodontitis, healthy individuals, and patients with complete prosthetics. Out of 15 positive samples, 86.7% were resistant to Co-amoxiclav, 73.3% Tetracycline and 13.3% Imipenem. These results show less resistance compared to our report (60% Tetracycline and 30% Imipenem). This difference cannot directly indicate an increase in resistance after a decade because other studies show a noticeable difference in the same period. [ 19 , 29 ] Another ten-year study from 2002 to 2011 at the Tunis Bone Marrow Transplant Center [ 19 ], 210 isolates of *P. aeruginosa* showed 32.8% resistance to Imipenem and/or Ceftazidime. The rate of resistance per 1000 days of illness increased from 2.07 to 2.21 [ 19 ]. This result is similar to the result (30%) obtained from our study.

Oliveira *et al.* [ 29 ] conducted a study in 2004 at 13 hematopoietic stem cell transplant centers in 10 Brazilian cities. A total of 411 patients with various types of cancer including myeloma and lymphoma were examined. Their results showed that 22% of isolates belonged to *P. aeruginosa*. Despite the results of our study, *P. aeruginosa* isolates were resistant to Imipenem (4/5), Ceftazidime (5/5), Ciprofloxacin (4/5), and Cefepime (5/5). One of the risk factors mentioned for multidrug resistance gram-negative bacteria was previous exposure to third-generation Cephalosporins and attending a specific transplant center.

A retrospective study by Agarwal and Larson [ 16 ] from 2012 to 2017 was conducted to assess the incidence of microbiological characteristics and risk factors associated with bacteremia in children candidates of hematopoietic stem cell transplantation. All Pseudomonas species isolated from the bloodstream showed resistance (1 case as multiple resistance and 4 cases as Carbapenem-resistant). A study in Libya [ 30 ] evaluated the antimicrobial susceptibility of *P. aeruginosa* in 46 patients admitted to the Burn and Plastic Surgery Center from 2013 to 2014. These patients had previously shown resistance to Ertapenem, Imipenem, and Meropenem. When comparing this study to our study, very high resistance of isolates of gram-negative bacilli against Carbapenem and multiple resistances in all isolates, including Gentamicin 45 (98%), Ceftazidime 42 (91%), Cefepime 39 (85%), and Ciprofloxacin 45 (98%) were observed [ 30 ].

A study of drinking water samples from various environments, including health centers in 2017 [ 31 ] reported that although Imipenem was still considered a front-line antibiotic against *P. aeruginosa*, the persistent increase in resistance was detected. This study emphasizes the role of purines as an effective factor in Carbapenem resistance in gram-negative bacteria [ 31 ]. In addition, another study identifies mutations that, among other reports, indicate a new resistance mechanism that may play a role in Imipenem resistance for *P. aeruginosa* [ 32 ].

Combination therapy can help maximize bacterial killing, minimize resistance, or both [ 33 ]. In the study of Kraiem *et al.* [ 30 ], only Colistin showed excellent performance against *P. aeruginosa*. However, the data showed that the center's staffs prevent the use of Colistin in the treatment of serious infections due to concerns about nephrotoxicity. The results of a similar study showed that a combination of Colistin and Amikacin might be effective in the treatment of Metallo-β-lactamases of Carbapenem-resistant gram-negative bacilli in serious life-threatening infections [ 29 ]. Although limited cases of Colistin resistance has been interpreted, the beginning of its resistance development has been announced [ 34 ]. An important issue that is being mentioned in literature nowadays is the effect of chemotherapy on enhancing antibiotic resistance. It is hypothesized that cancer chemotherapeutic compounds can damage bacterial DNA, which in turn enhances the bacterial mutation rate. Antibiotic resistance occurs after mutation of bacterial core genes. [ 35 ] Meunier *et al.* [ 35 ] showed that chemotherapy accelerates evolution of the microbiota and drive the emergence of antibiotic-resistant mutants from bacterial commensals in patients in an *in vitro* study. This matter also emphasizes the importance of antibiotic resistance studies in this group of patients. 

It is worthy to mention that salivary samples were chosen in this study due to smaller sample fraction, good patient compliance, easy collection (without specialized equipment or personnel), easy storage and transportation, cost effectiveness, greater sensitivity, and correlation with levels in blood [ 35 - 36 ]. In addition, as mentioned previously, this bacterium is isolated from the oral cavity and the saliva can be a non-invasive biomarker that can predict the susceptibility to pulmonary *P. aeruginosa* [ 11 - 12 , 36 ].

Moreover, it can be stated that the results obtained from most of the studies [ 15 - 16 , 19 , 29 - 30 ] mentioned were in line with the results of the present study. In some cases, differences in percentages may be rooted in differences in the number of study samples. One of the limitations of our study was the small sample size due to the COVID-19 pandemic, which did not allow further data to be collected. Besides, other factors such as site of sampling, which was saliva in the present study, can play a role in the different results seen with other studies. In most studies, open wounds and mucus were common sites of sample collection. The lack of a significant relationship between antibiotic resistance and age, gender, or type of disease was predictable due to the small statistical population. Future studies with larger sample sizes are suggested.

## Conclusion

The results of this study show the ineffectiveness of the Penicillin family against *P. aeruginosa*. Tetracycline and Sulfamethoxazole also did not show promising results. Cefotaxime, Gentamicin, Ceftazidime, and Cipro-floxacin and Cefepime were less resistant in the present study than in other studies. This may indicate the therapeutic potential of these drugs in Iran. Further research is needed to achieve more reliable results for previously mentioned antibiotics. None of the articles reviewed mentioned Trimethoprim and Vancomycin. The poor performance of Trimethoprim requires further study. However, Vancomycin along with its noticeable performance makes it more valuable for further research and use.

### Data Availability

The data supporting the findings of this study are available upon reasonable request from the corresponding author. However, restrictions were applied to the public availability of these data, because of the patient's confidentiality.
